# Managing acute presentations of atheromatous renal artery stenosis

**DOI:** 10.1186/s12882-022-02813-8

**Published:** 2022-06-16

**Authors:** Áine de Bhailis, Saif Al-Chalabi, Rodrigo Hagemann, Sara Ibrahim, Amy Hudson, Edward Lake, Constantina Chysochou, Darren Green, Philip A. Kalra

**Affiliations:** 1 Department of Renal Medicine, Northern Care Alliance NHS Foundation Trust, Salford, England; 2 Department of Vascular Interventional Radiology, Manchester University NHS Foundation Trust, Manchester, England

**Keywords:** Hypertension, Renal Artery Stenosis, Renal Revascularisation, Chronic, Kidney Disease, Heart Failure

## Abstract

**Background:**

Atherosclerotic renovascular disease (ARVD) often follows an asymptomatic chronic course which may be undetected for many years. However, there are certain critical acute presentations associated with ARVD and these require a high index of suspicion for underlying high-grade RAS (renal artery stenosis) to improve patient outcomes. These acute presentations, which include decompensated heart failure syndromes, accelerated hypertension, rapidly declining renal function, and acute kidney injury (AKI), are usually associated with bilateral high-grade RAS (> 70% stenosis), or high-grade RAS in a solitary functioning kidney in which case the contralateral kidney is supplied by a vessel demonstrating renal artery occlusion (RAO). These presentations are typically underrepresented in large, randomized control trials which to date have been largely negative in terms of the conferred benefit of revascularization.

**Case presentation:**

Here we describe 9 individual patients with 3 classical presentations including accelerated phase hypertension, heart failure syndromes, AKI and a fourth category of patients who suffered recurrent presentations. We describe their response to renal revascularization. The predominant presentation was that consistent with ischaemic nephropathy all of whom had a positive outcome with revascularization.

**Conclusion:**

A high index of suspicion is required for the diagnosis of RAS in these instances so that timely revascularization can be undertaken to restore or preserve renal function and reduce the incidence of hospital admissions for heart failure syndromes.

## Background

Atherosclerotic renovascular disease (ARVD) often follows an asymptomatic chronic course which may be undetected for many years. The prevalence of ARVD ranges depending on the population studied. In the general asymptomatic population, it is estimated that 6.8% of the population over the age of 65 years have evidence of 50% or greater renal artery stenosis (RAS) on duplex ultrasound [1]. In those with established cardiovascular disease or significant risk factors the prevalence increases to up to 54% in those with congestive heart failure [2].

However, there are certain critical acute presentations associated with ARVD and these require a high index of suspicion for underlying high-grade RAS to improve patient outcomes. These acute presentations, which include decompensated heart failure syndromes, accelerated hypertension, rapidly declining renal function, and acute kidney injury (AKI), are usually associated with bilateral high-grade RAS (> 70% stenosis), or high-grade RAS in a solitary functioning kidney in which case the contralateral kidney is supplied by a vessel demonstrating renal artery occlusion (RAO). As the latter presentations are rarely associated with flank pain or haematuria, the consensus is that these RAO are usually chronic.

In contrast, acute RAO is a rare and organ threatening condition with varied presentations. The potential causes include thromboembolic disease, renal artery thrombosis, dissection and iatrogenic during endovascular procedure. One of the leading causes of acute occlusion is atherosclerosis. The majority of patients with acute RAO present with flank pain or with features such as fever, nausea, uncontrolled hypertension, haematuria or acute renal injury [3]. Nonspecific features can result in a delay in diagnosis which leads to delayed management and long-term sequelae such as chronic kidney disease (CKD).

Functional MRI studies measuring tissue oxygenation by blood oxygenation level dependant (BOLD) MRI have shown that when increasing severity of RAS occurs beyond 70% there is risk of progressive reduction in perfusion leading to cortical ischaemia [4]. This in turn leads to reduced renal blood flow and oxygenation within the affected kidney, with consequent activation of inflammatory pathways with increased levels of biomarkers such as neutrophil gelatinase- associated lipocalin (NGAL) ultimately leading to fibrosis [5]. For optimal patient outcome a haemodynamically significant atheromatous lesion must be identified and addressed early before the inflammatory pathway is activated.

Our centre has specialised in the management of patients with ARVD for over 3 decades, treating over 1000 patients with the condition during this time. The vast majority of patients have a sub-acute or chronic presentation. These are the patient phenotypes highly represented in the large scale randomised control trials (RCT) of stenting and medical care versus medical care alone in ARVD, namely ASTRAL and CORAL, that failed to show a benefit of revascularization to outcomes of renal function, cardiovascular events and death. Acute presentations in patients with ARVD are much less common and such patient phenotypes only had minority representation in the RCTs. Expert opinion suggests that these patients are more likely to derive a positive benefit from renal revascularization procedures, and we wish to draw attention to the classical phenotypes which should not be overlooked by describing our experience of acute ARVD presentations over the last 15 years.

In this review we have described all patients referred to our institution with acute presentations of atheromatous RAS and have characterised their presentation, imaging, treatment, and long-term outcome. We describe 9 individual patients with 3 classic presentations including accelerated phase hypertension, heart failure syndromes, anuric AKI and a fourth category of patients who suffered recurrent presentations.

## Methods

The electronic health records of patients referred to Salford Royal for renal artery intervention between 2005 and 2020 were reviewed for diagnosis of “critical renal artery stenosis” or “renal artery occlusion”. Patients were included if they had complete occlusion of the renal artery or if they had an acute presentation with clinical features consistent with high-risk clinical phenotypes. This initially yielded 14 patients; 5 were excluded based on presence of trauma or other conditions such as vasculitis or fibromuscular dysplasia. In addition, their imaging in terms of CT or MR angiography was reviewed and patients were included if this confirmed the clinical suspicion of RAS which was deemed suitable for intervention. No MRA with gadolinium was performed in those with an eGFR of < 30 ml/min/1.73m^2^ to avoid risk of nephrogenic systemic fibrosis; contrast free MRA or CTA was performed in such patients. If CTA was indicated in those with eGFR of < 30 ml/min/1.73m^2^ a standard protocol of pre-hydration with normal saline given at a rate of 250 ml/hour for one hour pre-procedure and continued until one hour post CTA was employed to reduce the risk of contrast induced injury. Patients with ARVD without symptoms or features consistent with RAS were excluded. Data collected included demographics, co-morbidities, presentation, method of diagnosis, intervention, immediate outcomes, and long terms outcomes where available.

### Clinical presentations

In the 15 years from Jan 2005 to Jan 2020 there were a total of 156 percutaneous renal artery revascularisation procedures undertaken in 132 patients with atherosclerotic RAS within our unit. In this case series we reviewed those acute presentations treated with renal artery stenting; there were 16 such presentations in 9 individual patients.

#### 1) Accelerated phase hypertension

Accelerated hypertension is a hypertensive emergency characterised by systolic blood pressure > 180 mmHg and/or diastolic blood pressure > 120 mmHg with evidence of acute target organ damage [6]. RAS is a leading cause of secondary hypertension and renal ischaemic disease [7]. The belief for many years was that by restoring blood flow to a kidney affected by an atherosclerotic lesion would in essence cure hypertension. Several large randomised control trials have effectively shown that revascularisation does not confer a substantial benefit over medical treatment in those with renal artery disease [8,9]. However, as indicated earlier, high risk presentations were under-represented in these trials.

HERCULES ( A safety and effectiveness study of the Herculink Elite Renal Artery Stent to treat Renal Artery Stenosis), a prospective multicentre trial of 202 patients with ARVD undergoing intervention for uncontrolled hypertension, found a significant reduction in systolic blood pressure (SBP) in stent-treated patients with uncontrolled hypertension. The absolute reduction was related to severity of hypertension pre intervention, those with a starting SBP > 180 mmHg experiencing a mean fall of 48 mmHg while those with a starting SBP of 140–160 mmHg experiencing a mean reduction of 23 mmHg at 9 months [10]. This trial highlighted that when patients are appropriately selected for intervention, they can experience significant reductions in blood pressure and in the number of anti-hypertensive medications required for treatment. This is certainly in keeping with the experience of our unit in relation to revascularisation for the indication of severe/accelerated hypertension in the setting of RAS.

A group in Lyon with a similar MDT approach to those diagnosed with atherosclerotic renal artery disease between 2013 and 2015 who primarily focused on blood pressure control found better control in those assigned to intervention. Those in the revascularisation group had a decrease in systolic blood pressure of -23 ± 34 mmHg ( p = 0.007) and diastolic blood pressure of 12 ± 18 mmHg ( p = 0.007). There was also a reduction of antihypertensive agents and the number of cases of resistant hypertension at one year follow up. There was no change in those assigned to medical treatment [11].

Our unit is a dedicated renal centre and it is possible that accelerated phase hypertension treated by other specialities such as cardiology may be under represented in this case series. There were 3 patients whose initial presentation was that of accelerated phase hypertension (Table 1). All 3 patients responded well to intervention and achieved target blood pressure within one month of intervention. Of note one patient’s severe left ventricular systolic dysfunction improved significantly from an ejection fraction of 24% to 55%.

**Table 1:** Clinical Information of patients presenting with accelerated phase hypertension

Patient	Presentation	Comorbidities	Clinical Features	Anatomy	Intervention	Response
2	55 yr old male referred for resistant htn	HTN, IHD, Previous TIA PVD, Smoker	BP: 152/105 mmHg on 3 agents with hypertensive retinopathy	MRA Rt: 90% (10.5 cm) L: 60% (10.5 cm)	Rt PTRAS (Fig. 1)	1 month: BP < 130/80 mmHg on 3 agents
6	57 yr female referred for resistant htn	HTN (age 20), Asthma Chronic pain syndrome	BP: 204/101 mmHg on 5 agents	MRA Rt: 90% L: 100% (< 7)	Rt PTRAS	1 month: BP < 130/80 mmHg on 3 agents
8	63 yr old male referred for resistant htn	HTN, COPD, Smoker HFrEF (24%)	BP: 201/107 mmHg on 5 agents	MRA Rt: 100%(7 cm) L: > 70% (10 cm)	L PTRAS	1 month: BP < 130/80 mmHg on 3 agents 4 months: EF ↑ 55%

*HTN* hypertension, *IHD* ischaemic heart disease, *TIA* transient ischaemic attack, *PVD* peripheral vascular disease, *BP* blood pressure, *MRA* Magnetic Resonance Angiography, *Rt* right, *L* left, *PTRAS* percutaneous transluminal renal angioplasty with stenting, *COPD* chronic obstructive pulmonary disease, *HFrEF* heart failure with reduced ejection fraction, *EF* ejection fraction

**Fig. 1:**
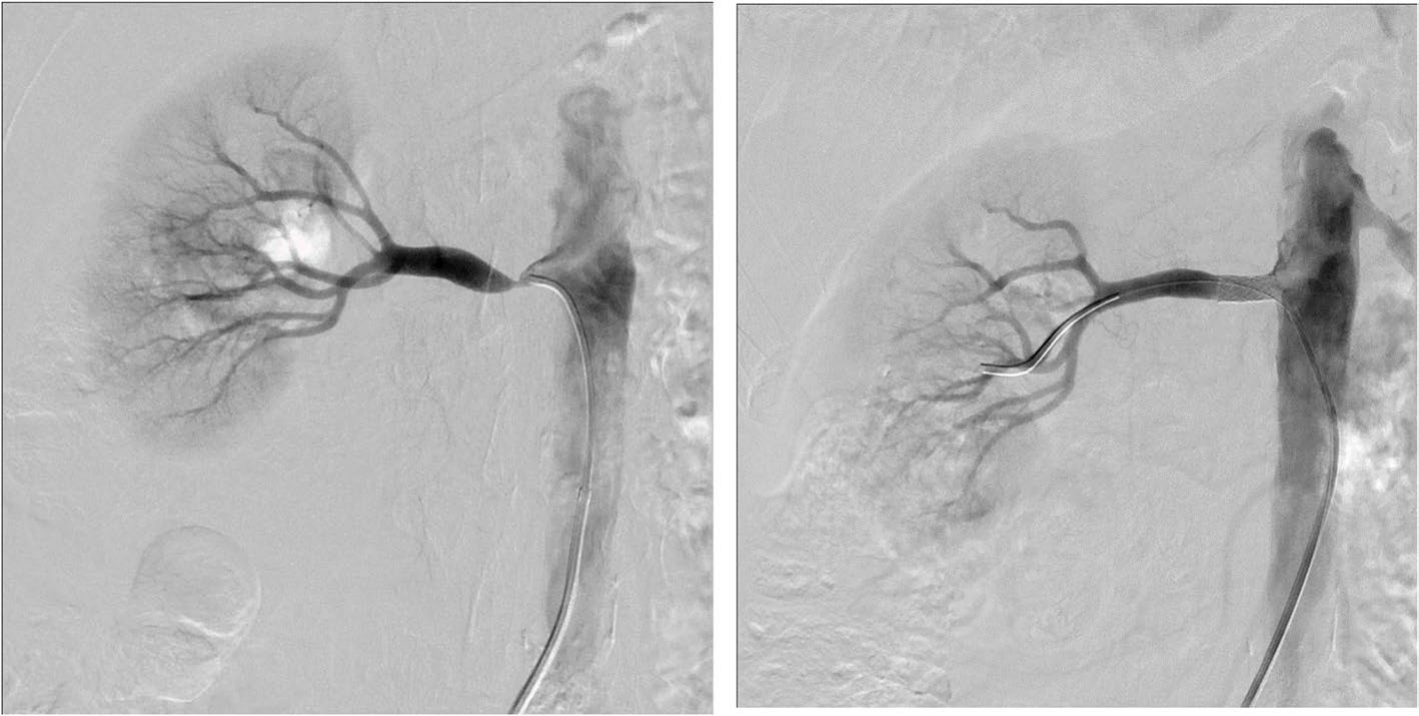
Left: Rt renal artery stenosis presenting as severe hypertension (Patient 2). Right: Successful Rt PTRAS with immediate improvement

#### 2) Heart failure syndromes

Sustained hypertension and the salt and water volume retaining state of atherosclerotic RAS play a role in the decompensation of patients with ARVD and heart failure. The hallmark ‘flash pulmonary oedema’ is a well-recognised manifestation of renal artery disease. Flash pulmonary oedema (or Pickering syndrome) is a general term used to describe a hyper-acute presentation of decompensated heart failure due to an acute rise of left ventricular end diastolic pressure.Although it has become synonymous with bilateral severe RAS or severe RAS affecting a solitary kidney, prompting the physician to consider screening for this underlying arterial pathology [12], it can also present in patients with other conditions such as acute coronary syndrome.

Previous reports from this unit have illustrated that patients presenting with heart failure syndromes, such as flash pulmonary oedema, if managed medically, have an increased risk for death and cardiovascular events but not for progression to end stage kidney disease. Revascularisation in these patients was associated with reduced risk of death but not CV events or ESKD [13].

Below (Table 2) is a description of two patients who presented to out unit with a clinical syndrome consistent with acute heart failure during the 15 year period. Their clinical presentation differs in terms of recurrent admission for heart failure versus first episode of acute heart failure however both had evidence of bilateral renal artery disease which responded well to revascularisation. Unfortunately, the second patient remained dialysis dependant and had several other acute events in the vascular beds of other organs.

**Table 2:** Clinical Information of patients presenting with heart failure

Patient	Presentation	Co-morbidities	Clinical Features	Anatomy	Intervention	Response
3	66 yr female referred for recurrent heart failure. 3 admissions in 12 months	HTN (poorly controlled), Progressive CKD (eGFR: 34)	BP: 193/93 mmHg on 5 agents	MRA Rt: 80–90% (9 cm) L: 80–90% (11 cm)	Bilateral PTRAS	1 month: Bp: 134/69 mmHg on 3 agents eGFR: 45 ml/min 4 months: EF: 45–55% No further admissions for HF
7	68 yr male acute presentation with dyspnoea while mobilising	HTN (> 20 years), Inflammatory arthropathy, Ex-smoker	Grossly oedematous and in pulmonary oedema Creatinine: 538 µmol/L; needed acute dialysis	CTA Rt: > 90% (11 cm) L: > 90% (9.5 cm)	Bilateral PTRA with left PTRAS	1 month: Remained HD dependant. NSTEMI not for PCI. 6 months: Visual loss due to retinal artery occlusion. PVD with acute limb ischaemia requiring angioplasty. 24 months: RIP

*HTN* hypertension, *CKD* chronic kidney disease, *eGFR* estimated glomerular filtration rate, *BP* blood pressure, *MRA* Magnetic Resonance Angiography, *Rt* right, *L*-left, *PTRAS* percutaneous transluminal renal angioplasty with stenting, *EF* ejection fraction, *HF* heart failure, *CTA* computed tomography angiography, *PTRA* percutaneous transluminal angioplasty, *HD* haemodialysis, *NSTEMI* non-ST segment elevation myocardial infarction, *PCI* percutaneous coronary intervention, *PVD* peripheral vascular disease

#### 3) Rapidly declining renal function and AKI

Ischaemic nephropathy is a term which describes a loss in renal mass with replacement fibrosis as indicated by a size discrepancy (due to renal atrophy) and/or significant proteinuria, and most patients have CKD. Previous non-randomised studies have indicated that those with rapidly progressive renal impairment are most likely to benefit from prompt revascularisation due to a degree of reversibility of renal function [14]. The patients less likely to benefit are those with proteinuria > 1 g/24 h, renal length < 7 cm, or those receiving renal replacement therapy for more than 3 months [15].

In patients with known atherosclerotic renovascular disease or those with evidence of generalised atheroma ( such as carotid bruit etc.) who present with AKI and no evidence of obstruction on imaging clinicians should have a high suspicion for this diagnosis. Early confirmation and intervention is indication which can transform outcomes for the affected individual.

Acute kidney injury is the most common acute presentation in the experience of our unit. Four of our 9 acutely presenting patients had this initial presentation, and it accounted for 5 of the recurrent presentations. All 4 patients presented with severe AKI requiring renal replacement therapy who recovered significant function post intervention and remained independent of dialysis. (Table 3).

**Table 3:** Clinical information of patients presenting with rapidly decline renal function/AKI

	Presentation	Co-morbidities	Clinical Features	Anatomy	Intervention	Outcome
1	56 yr female referred from another unit with AKI, volume overload and htn	Diastolic dysfunction HTN ( 4 agents) PVD with claudication distance of < 20 m CKD (eGFR 29 ml/min) Smoker	BP: 189/90 mmHg Creatinine: 1017 µmol/L (AKI on CKD) with hyperkalaemia and metabolic acidosis	MRA Rt: 100% (8.5 cm) L: > 80% (10.5 cm)	LPTRAS	Immediate ↑ in UO (1.5L/24 h) and ↓ creatinine to 598 µmol/L 1 month: Creat 115 µmol(eGFR: 44 ml/min) BP: 149/70 mmHg on 2 agents RIP 7 years later: no further presentations
4	78 yr female transferred from another institution to our unit with accelerated phase hypertension, deteriorating renal function and pulmonary oedema	HTN with LVH NIDDM CKD (eGFR 40 ml/min) History of temporal arteritis PVD Diverticular disease Renal adenocarcinoma requiring L nephrectomy	Bp: 157/80 mmHg on iv diuretic infusion and 4 agents Volume overloaded Creatinine: 519 µmol/l (eGFR: 7 ml/min)	MRA Rt: > 90% (11 cm)	Rt PTRA	Within 1 week: BP < 130/80 mmHg on 2 agents Independent of RRT with creatinine of 128 µmol/L
5	63 yr male transferred for AKI on CKD	CKD 3 (ARVD within previous L PTRAS in 2012) HTN Previous ischaemic stroke NIDDM Hyperthyroidism treated with radioactive iodine therapy Smoker	BP: 198/92 mmHg on 3 agents Creatinine: 319 µmol/L(eGFR: 9 ml/min) uPCR: 800 mg/mol	Formal Angiogram: Occluded L stent	L PTRAS (Fig. 2)	Within 1 month: BP: < 130/80 mmHg on no agents Creatinine:240 µmol/L (eGFR: 24 ml/min) uPCR: 132 mg/mol
9	74 yr female presented acutely with anuric AKI	HTN IHD with angina Macular degeneration Smoker	BP 190/90 mmHg Volume overloaded Creatinine: 633 µmol/L (eGFR: 6 ml/min)	Renal US: Rt: 11 cm L: 8.2 cm Formal Angiogram: Rt: > 70% L: 100%	Rt PTRAS	Within 1 month: BP: < 130/80 mmHg on single agent Creatinine: 150 µmol/L (eGFR: 31 ml/min)

*AKI* Acute Kidney Injury, *HTN* hypertension, *PVD* peripheral vascular disease, *CKD* chronic kidney disease, *eGFR* estimated glomerular filtration rate, *BP* blood pressure, *MRA* Magnetic Resonance Angiography, *L* left, *PTRAS* percutaneous transluminal renal angioplasty with stenting, *UO* urinary output, *LVH* left ventricular hypertrophy, *NIDDM* non insulin dependent diabetes mellitus, *PTRA* percutaneous transluminal renal angioplasty, *RRT* renal replacement therapy, *ARVD* atheromatous renovascular disease, *uPCR* urine protein to creatinine ratio, *IHD* ischaemic heart disease, *US* ultrasound, *Rt* right

**Fig. 2:**
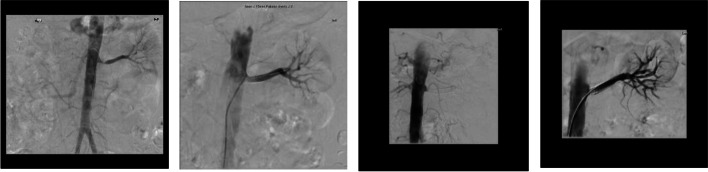
(from left to right). 1) L RAS presenting as severe hypertension. 2) Successful L PTRAS with BP on target with single agent. 3) 5 years later LRAS presenting as anuric AKI. 4) Successful L PTRAS with immediate urinary output. (Patient 5)

### Recurrent presentations

Several case series exist describing revascularisation for the management of occlusive renal artery disease. Lawrie, Morris and DeBakey described their experience of 40 patients with renovascular hypertension and totally occluded renal arteries in the 1980s, before widespread application of percutaneous revascularization techniques. 21 of these patients underwent surgical renal artery reconstruction, 15 of which were successful which was defined by long term patency (mean of 223 months). This study assessed potential factors predictive of a good outcome including sex, age, renal function, contralateral reconstruction, technical factors and physical attributes of affected kidney and only size and weight of kidney were consistent predictors of outcome [16]. Whitehouse et al. also reported similar experience based on 40 patients with hypertension [17]. However, there is little in the literature in terms of experience with recurrent presentations of acute RAS.

A large case series of 363 patients undergoing renal artery stenting for either hypertension or renal insufficiency included follow up in which re-stenosis was found in 21% of 102 patients overall; this was less common in large calibre vessels ie > 4.5 mm [18]. The majority (87%) of these patients had non-acute presentations with hypertension at baseline as the indication for intervention.

Four of our 9 patients had recurrent presentations, with 11 presentations between them (Table 4). Their subsequent presentations often differed from their original clinical picture, but the predominant presentation was that of ischaemic nephropathy, with5 of the 11 recurrent presentations being AKI and all but one responded well to intervention. One patient remained dialysis dependent, but it is important to note in this subject there was a technical failure to cross the RAS lesion to deploy a stent.

**Table 4:**
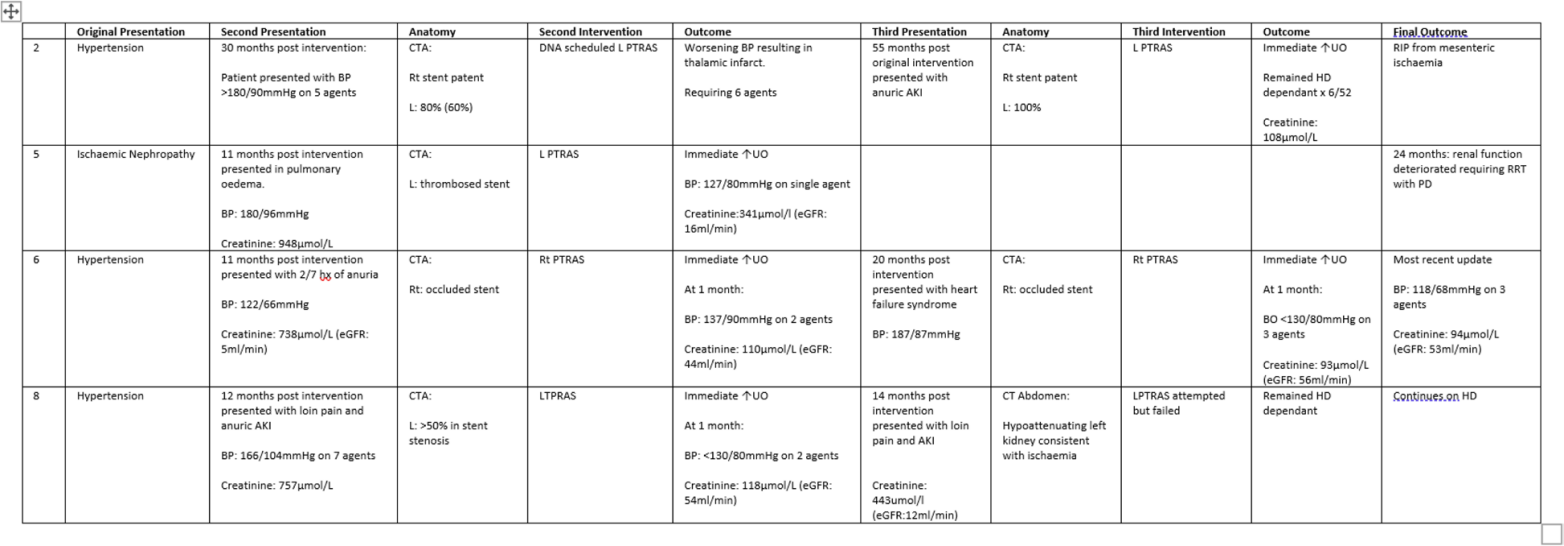
Clinical information for patients with recurrent presentations

*BP* blood pressure, *CTA* computed tomography angiography, *DNA* did not attend, *L* left percutaneous transluminal renal angioplasty with stenting, *AKI* Acute Kidney Injury, *UO* urinary output, *RRT* renal replacement therapy, *PD* peritoneal dialysis, *Rt* right, *eGFR* estimated glomerular filtration rate, *CT* computed tomography, *HD* haemodialysis

## Discussion

The 16 acute presentations of atherosclerotic RAS, treated by renal stenting, which are described in this report equated to 10% of all revascularisation procedures performed in our patients, highlighting that acute presentations are relatively uncommon (in our experience an average of one case per year in a total referral population of > 1 million) and that they necessitate a high index of suspicion and timely intervention.

All of the RAS lesions were detected by CTA or MRA, techniques which have high sensitivity and specificity for detecting significant RAS. Doppler ultrasound is also an important diagnostic and screening tool for evaluating RAS with a sensitivity and specificity for diagnosing significant RAS up to 90% and 95% respectively [19]. It is acknowledged that duplex ultrasound requires significant operator time and is not currently available in many UK centres including our own.

All our patients were revascularized using percutaneous techniques involving RAS dilatation and stent placement. Prior to availability of angioplasty surgical repair was the only option for correction of atherosclerotic renal artery disease. In modern practice percutaneous intervention has replaced surgical intervention for the management of most cases of RAS due to the associated morbidity and mortality risk of surgery. Surgery is now reserved for more complex anatomical cases, such as those with multiple small renal arteries or where aortic reconstruction is required at the origin of the renal arteries in association with aneurysm repair, where severe aortoiliac disease co-exists, and in patients in whom stenting procedures have failed [20].

The predominant acute presentation was that of acute ischaemic nephropathy as manifest by AKI or deteriorating renal function, accounting for 56% (9/16) of the presentations. Accelerated hypertension accounted for 25% (4/16) of cases with heart failure accounting for 19% (3/16) of cases. Although the characteristic presentation of acute RAO is a patient presenting with loin pain and anuric AKI this classic presentation was seen on just 2 occasions and in only one individual patient, and only in subsequent presentations rather than the original one.

Severe hypertension accounted for 25% of our acute presentation cases. Hypertension is a common co-morbidity worldwide and a leading cause of mortality. Prevention and control of hypertension decreases mortality and incidence of heart failure. The prevalence in the past two decades has been increasing possibly due to increased consumption of processed food and changes in lifestyle [21]. Up to 15% of those with hypertension will develop resistant hypertension [22].

In the general population there is a high prevalence of resistant hypertension amongst those with CKD with increasing prevalence seen with reducing renal function, ranging from 15% in those with eGFR ≥ 60 ml/min/1.73m^2^ to 33.4% in those with eGFR < 45 ml/min/1.73m^2^ [23]. Previous RCT such as STAR, CORAL and ASTRAL [8, 24,25] have failed to demonstrate a significant benefit to patient outcome in patients with poorly controlled blood pressure or progressive renal impairment.

Other smaller studies of patients with high grade RAS that was managed medically without revascularisation found that more anti-hypertensive medications were required to effectively control blood pressure and that 5.8% of patients eventually required revascularisation [26]. In our case series, those with severe hypertension were receiving an average of 5 anti-hypertensive agents before stenting, which improved to an average of 3 agents to achieve target blood pressure post revascularisation. Our case series emphasises the need for monitoring patients with hypertension and other vascular risk factors such as atherosclerosis, smoking and dyslipidaemia to identify those most likely to benefit from revascularisation so as to reduce morbidity and mortality.

Previous studies have highlighted the benefit of revascularisation in those with heart failure with a substantial reduction in all-cause mortality and hospital admission [27]. The presence of an acute heart failure syndrome was the indication for revascularisation in 19% of the acute RAS presentations to our unit. In two of these patients there was a significant improvement in volume overload, blood pressure and renal function. One of the patients who did not benefit from stenting had previously benefitted from revascularisation on two separate occasions. There is a high prevalence of RAS amongst those with more chronic congestive heart failure ranging from 15 to 54% depending on the cohort studied and imaging modality used to identify the presence of RAS. In those with heart failure the presence of RAS is associated with worse renal outcomes and higher mortality [28].

**Fig. 3:**
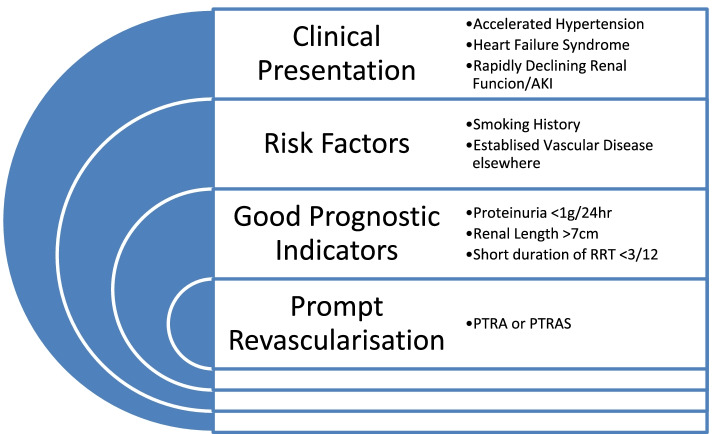
Key Messages with Acute presentations of atherosclerotic RAS

We believe that three important considerations are necessary when contemplating renal revascularization for patients with atherosclerotic RAS. The clinical presentation is key but so too is the severity of the RAS lesion and the extent of parenchymal damage within the kidney (with better results anticipated when there is minimal proteinuria and absence of significant renal atrophy). Our case series highlighted cases compatible with the recommendations of the ACC/AHA 2018 guidelines that suggest revascularisation should be reserved for those patients with “high risk” presentations such as recurrent flash pulmonary oedema, rapidly declining renal function and refractory hypertension [29] (Fig. 3).

## Conclusion

Acute presentations of atheromatous RAS are relatively uncommon but should be considered in patients presenting with severe hypertension, acute heart failure syndromes or with rapidly deteriorating renal function, especially AKI, particularly if there is evidence of widespread atheromatous disease. A high index of suspicion is required for the diagnosis of RAS in these instances so that timely revascularization can be undertaken to restore or preserve renal function and reduce the incidence of hospital admissions for heart failure syndromes. These are situations in which targeted early renal revascularization has a genuine chance of improving survival.

## Data Availability

Not applicable.

## Abbreviations

ARVD: Atherosclerotic renovascular disease
RAS: Renal artery stenosis
AKI: Acute kidney injury
RAO: Renal artery occlusion
CKD: Chronic kidney disease
BOLD: Blood oxygenation level dependent
MRI: Magnetic resonance imaging
NGAL: Neutrophil gelatinase associated lipocalin
ASTRAL: Angioplasty and Stenting for Renal Artery Stenosis
CORAL: Cardiovascular Outcomes in Renal Atherosclerotic Lesions
CT: Computed Tomography
HERCULES: The Safety and Effectiveness Study of the Herculink Elite Renal Stent to Treat Renal Artery Stenosis trial
SBP: Systolic blood pressure
MDT: Multi-disciplinary team
HTN: Hypertension
IHD: Ischaemic heart disease
TIA: Transient ischaemic disease
PVD: Peripheral vascular disease
BP: Blood pressure
MRA: Magnetic resonance angiography
CTA: Computed tomography angiography
Rt: Right
PTRA(S): Percutaneous transluminal renal angiography (with stenting)
COPD: Chronic obstructive pulmonary disease
HFrEF: Heart failure with reduced ejection fraction
EF: Ejection fraction
CV: Cardiovascular
ESKD: End Stage Kidney Disease
eGFR: Estimated glomerular filtration rate
HD: Haemodialysis
NSTEMI: Non ST segment elevation myocardial infarction
PCI: Percutaneous coronary intervention
UO: Urinary output
NIDDM: Non insulin dependent diabetes mellitus
RRT: Renal replacement therapy
uPCR: Urine protein creatinine ratio
US: Ultrasound
DNA: Did not attend
PD: Peritoneal dialysis
